# Genetically unmatched human iPSC and ESC exhibit equivalent gene expression and neuronal differentiation potential

**DOI:** 10.1038/s41598-017-17882-1

**Published:** 2017-12-13

**Authors:** Hany E. Marei, A. Althani, S. Lashen, C. Cenciarelli, Anwarul Hasan

**Affiliations:** 1 Biomedical Research Center, Qatar University, Doha, PO Box 2713, Qatar; 2 Department of Health Sciences, College of Health Sciences, Qatar University, Doha, 2713 Qatar; 3 Department of Cytology and Histology, Faculty of Veterinary Medicine, Mansoura University, Mansoura, Egypt; 4 Institute of Translational Pharmacology-CNR, Roma, Italy; 5 Department of Mechanical and Industrial Engineering, Qatar University, Doha, Qatar

## Abstract

The potential uniformity between differentiation and therapeutic potential of human embryonic stem cells (hESCs) and human induced pluripotent stem cells (hiPSCs) remains debatable. We studied the gene expression profiles, pathways analysis and the ability to differentiated into neural progenitor cells (NPCs) and motor neurons (MNs) of genetically unmatched integration-free hiPSC versus hESC to highlight possible differences/similarities between them at the molecular level. We also provided the functional information of the neurons derived from the different hESCs and hiPSCs lines using the Neural Muscular Junction (NMJ) Assay. The hiPSC line was generated by transfecting human epidermal fibroblasts (HEF) with episomal DNAs expressing Oct4, Sox2, Klf4, Nanog, L-Myc and shRNA against p53. For the hESCs line, we used the NIH-approved H9 cell line. Using unsupervised clustering both hESCs and hiPSCs were clustered together implying homogeneous genetic states. The genetic profiles of hiPSCs and hESCs were clearly similar but not identical. Collectively, our data indicate close molecular similarities between genetically unmatched hESCs and hiPS in term of gene expression, and signaling pathways. Moreover, both cell types exhibited similar cholinergic motor neurons differentiation potential with marked ability of the differentiated hESCs and hiPSCs-derived MNs to induce contraction of myotubes after 4 days of co-culture.

## Introduction

Although human embryonic stem cells (hESCs) are considered to be the “gold standard” of pluripotent cell lines, ethical concerns regarding the way in which they are generated limit their clinical applications. Successful generation of human induced pluripotent stem cells (hiPSCs) by overexpression of the transcription factors Oct4, Klf4, Sox2 and c-Myc could provide promising substitute for hESCs. These substitutes would overcome ethical and moral issues associated with the use of hESCs in the preclinical and clinical settings.

Having two well-established sources of pluripotent cell lines (hESCs and hiPSC), it is critically important to elucidate whether or not they are identical/equivalent. The presence of fundamental differences between the transcriptomes of hiPSCs and hESCs have been reported^1^. Using larger numbers of samples, both cell lines have been shown to be nearly identical^2,3^. The use of recent platforms for studying the gene expression profiles of both cell types has revealed small numbers of differentially modulated genes^4^. Several confounding factors have been suggested as causal factors generating the differences in gene expression profiles of hiPSCs and hESCs: these are reprogramming methods^5^, host genetic background^6^, clonal origin^7^, gene silencing^8^ and sex of cell lines^9^. One possible way to decipher the effects of different host genetic backgrounds on gene expression, and neuronal differentiation potential of hiPSCs and hESCs is to eliminate major confounding factors such as reprogramming method by comparing genetically unmatched ESCs and integration-free iPSCs.

Traditionally, somatic and pluripotent cells are distinguished from each other using PCR and immunostaining to detect specific well-characterized biomarkers^10,11^. Such an approach may produce inaccurate findings due to instabilities within inherent pluripotent cell lines^11^. It has been reported that the OCT4 biomarker, a reliable marker to differentiate ESCs from somatic cells, is not consistently expressed in older ESCs. Moreover, low abundance marker protein signals are difficult to detect using current antibody based biomarkers due to low sensitivity of this technique^11^.

Although a combined array of linear models, cluster analysis and gene expression profiling can be used to distinguish hiPSCs from hESCs^12^, a distinction between hESCs from hiPSCs based only on gene expression and cluster analysis is challenging since the gene signature is not consistent across different cell lines^13,14^. Furthermore, in most cases cluster analysis is associated with a low level of sensitivity in the presence of different cellular sources^14^. There is therefore still a need to design an approach that overcomes these limitations so as to be able to accurately discriminate between the closely similar pluripotent hiPSCs and hESCs.

Linking gene expression with the ability to differentiate into specific cell lines (such as neuronal lineage) as well as providing functional information of the neurons differentiated from different iPSCs and hESCs may be a promising approach to discriminate between hiPSCs and hESCs. We explored the potential differences in gene expression profiles between genetically unmatched, integration-free hiPSCs and NIH-approved H9 hESCs using cluster analysis, gene ontology, biological pathways, their ability to differentiate into NPCs and NMJ Function Assay (Contractility Assay) of the generated iPSCs and hESCs-derived MNs, to elucidate the degree of gene expression uniformity, potential neuronal differentiation potential, and functional information of the neurons differentiated from the two cell populations. Such an approach would help to eliminate major confounding factor related to the presence of integrating DNA viruses during the generation of iPSCs. Provision of a proof of principle determining potential molecular uniformity between genetically unmatched hESCs and hiPSCs would ensure similar differentiation potential and functional outcomes which in turn could expedite the application of hiPSCs in the clinical setting.

## Methods

### Ethical statement

All cell lines including hESC and iPSC were obtained commercially from IXtechnologies, USA (http://www.ixcellsbiotech.com). The experimental protocol had obtained an IRB waiver from Qatar University Inistitutional Review Board (IBR) due to the use of commercial cell line with no involvement of any sample collections from human.

#### Generation of human iPSCs

Human dermal fibroblasts were transfected with episomal DNAs expressing Oct4, Sox2, Klf4, Nanog, L-Myc and shRNA against p53 15. The transfected cells were seeded on CF1 MEF feeder plates and maintained in human iPSC derivation media for 4–5 weeks. The iPSC colonies were collected and transferred to MEF feeder plates for further expansion. The fibroblasts, iPSCs, and hESC were derived from males. One of the used hESC lines was derived from female. The transfection efficiency was about 30–50% (~100–200 iPSC colonies were obtained). Both hESCs and iPSCs were maintained in mTeSR1 media (Stem Cell Technologies) on Matrigel-coated plates.

#### Expansion and cryopreservation of human iPSCs

The iPSC clones were maintained on MEF feeder plates for one passage and switched to mTeSR1 media without MEF beginning with the second passage. The cells were passaged every 5–7 days until cryopreservation and cell pellet preparation (for downstream assays, e.g. PCR, antibody staining, flow cytometry, microarray, NMJ Assay etc).

#### Quality Control of End of Production cells

For pathogen screening, the cells were screened to detect any risk of bacterial, fungal and/or mycoplasmal contamination. For purity testing, antibody staining (ICC) and flow cytometry (IF) using cell-type specific antibodies were used. For an episomal DNA integration assay, Q-PCR allowed the quantitative determination of the copy number of episomal DNA integrated into the cells. A cell was considered integration-free if less than 0.01 copy of episomal DNA was detected. For karyology, metaphases were examined for chromosome number and banding pattern. Twenty cells were analyzed.

#### RNA extraction

hiPSCs and hESCs were pelleted, and resuspended in TRIZOL at a volume of 5 × 10^6^ cells/1 mL TRIZOL. 200 µl chloroform was added to each tube for 5–8 minutes of incubation. Samples were centrifuged at 12,000 g for 15 minutes at 2–8 °C followed by RNA precipitation using isopropanol. RNA pellets were washed and treated with Dnase before loading on an Rneasy column (Qiagen) for final purification. RNA quantification and quality control were conducted using Gel electrophoresis based on OD values. The RIN software algorithm was used accurately classify the samples. All samples with a RIN higher than the threshold value pass the QC test, while samples below the threshold value are discarded. The cut-off or threshold for RNA quality was adjusted to RIN 8%^15^.

#### Microarray

The RNA quantity was analyzed using the Nano Drop ND1000 (SOP Nu TAL009) and the RNA quality is checked using the Bioanalyzer 2100 (Agilent). Sample amplification was performed with 200 ng of total RNA using Agilent’s Quick Amp Labeling Kit OnoColor to generate complementary RNA (cRNA) for oligo microarrays. cRNA was processed for microarray analysis on a Whole Human Genome Oligonucleotide Microarray (G4112A, 41,000 genes; Agilent Technologies, Santa Clara, CA, USA, and Illumina, USA), according to the manufacturer’s instructions.

The arrays were hybridized at 60 °C for 17 h with the Tecan HS Pro hybridization station in the hybridization buffer containing fluorescence-labeled cRNA. The microarrays were washed once with 63 SSPE buffer containing 0.005% N-laurylsarcosine for 1 min at room temperature followed by a second wash with pre-heated 0.066 SSPE buffer (37 °C) containing 0.005% N-laurylsarcosine for 1 min. The last washing stage was performed with acetonitrile for 30 sec.

Fluorescence signals of the hybridized microarrays were detected using Agilent’s and Illumina DNA microarray scanner (Agilent and Illumina Technologies) with a resolution of 5 lM. Agilent Feature Extraction Software (FES) was used to read out and process the microarray image files. The software determines feature intensities and normalized ratios by linear LOWESS with background subtraction, rejects outliers and calculates statistical confidences (P-values). A quality control was performed at this stage. Genes with a P-value smaller than 0.001 were considered significant. Only genes differentially expressed in all experiments were considered as relevant genes.

#### Data processing

Signal intensities were normalized by Cubic Spline strategy in the GenomeStudio software to reduce differences due to technical variation (false positives), while conserving true biological effects (i.e., maximizing true positives and minimizing false negatives)^16^. Probes returning a p-value < 0.05(t-test) and fold change(FC) > 2 in comparisons of the control and test classes were considered to be detecting differential expression. To avoid distortion of the results by noise, we removed probes returning signals that were highly likely to be due to non-specific background signal rather than specific probe-target hybridization. The specificity of individual probe signals was estimated using the detection p-value, which is the probability of seeing a certain signal level without probe-target hybridization. All probes returning a detection p-value > 0.01 in both the control and test classed were eliminated from further analysis^16^. Genes with significant difference in expression levels were uniquely retrieved from all probes.

All significantly expressed genes (P < 0.05, FC > 2, detection P < 0.01) were clustered using a hierarchical clustering method. Data from each sample were normalized in log-space to have a mean of zero using Cluster 3.0 software.

#### Gene Ontology Analysis

Gene ontology (GO) analysis was conducted according to published methods^17–19^. We considered a GO category significantly differentially regulated if either significance level was less than 0.01.

#### Functional Annotation and Molecular Network Analysis

Functional annotation of significant genes identified by microarray analysis was performed by the Database for Annotation, Visualization and Integrated Discovery (DAVID) version 2009 from the National Institute of Allergy and Infectious Diseases (NIAID), National Institutes of Health (NIH) (david.abcc.ncifcrf.gov)^20–23^. Gene ontology (GO) and KEGG molecular pathway analysis were performed to identify possible enrichment of genes with specific biological themes using both the data set as a whole and then in the individual K-means clusters.

#### Differentiation of Human iPSC/ESC to neural progenitors cells (NPC) and motor neuron (MN)

The iPSC and ESC were differentiated to generate ESC/iPSC derived NPC and MN and were subjected to transcriptomic analysis by qPCR on top 10 induced and suppressed genes identified in by DNA microarray. A list of primers used for the top 10 induced/repressed genes is provided in Supplementary Table 1. Each differentiation step were confirmed by immunostaining and flow cytometry (NPC: nestin; MN: CHAT). mRNA extracted from iPSC/ESC, NPCs, MNs are subjected to RT-PCR for the profiling of the genes listed above.

#### NPC differentiation

ESCs (H9) and iPSCs were cultured in neural progenitor differentiation medium (iXCells Biotechnologies, USA). The neural progenitor cells (NPCs) were characterized by immunofluorescence staining (Nestin) and flow analysis (Nestin), and also collected for RNA extraction.

#### For MN differentiation

NPCs derived from ESCs or iPSCs were further differentiated into mature motor neurons using the proprietary protocol (iXCells Biotechnologies). The motor neurons (MNs) were characterized by immunofluorescence staining (ChAT, MAP2) and flow analysis (ChAT), and also collected for RNA extraction.

#### NMJ Function Assay (Contractility Assay)

C2C12 mouse myofibroblasts were seeded on 96-well plates in DMEM with 10% FBS, and myotube differentiation were initiated by switching to DMEM with 2% FBS for 3–4 days. The iPSC-MNs or ESC-MNs were seeded on top of the differentiated myotubes with Motor Neuron Maintenance Medium (iXCells, Cat# MD-0022). The MN-dependent contraction of myotubes was observed after 4 days of co-culture.

#### RT-PCR

To confirm the DNA microarray results, we studied the gene expression profile for the top ten induced/repressed genes between hiPSCs/hESCs. The RNA was extracted using Trizol Reagent. For each sample, 1 µg RNA was used for cDNA synthesis using Transcriptor Universal cDNA Master (Roche, Cat# 05893151001). The real-time PCR was performed using FastStart Essential DNA Green Master (Roche, Cat# 06924204001) with triplicates derived from different cell lines.

#### Immunostaining

hiPSCs/hESCs-derived cell NPCs/MN were fixed in 4% paraformaldehyde and permeabilized with 0.2% Triton-100 (this last step was not performed for NG2 and O4 immunostaining) and subsequently processed for immunolabeling. The following antibodies were used: α-hTRA-1–81 (330704, BioLegend); α-hCD90 (328118, BioLegend); α-OCT4 (ASK-3006, Applied StemCell); α-PAX6 (cat. no. PAX6, DSHB); α-SOX1 (cat. no. 4194, Cell Signaling); PAX6 (cat. no. 5790, Abcam); GAPDH (cat. no. 2118, Cell Signaling); β-actin (cat. no. MA5-15739-HRP, Thermo Scientific).

#### Statistical analysis

Statistical analysis was performed using Two-way ANOVA and Bonferroni’s posttests. Data are expressed as mean ± standard error of mean (S.E.M). P values between < 0.05 (*) and < 0.001 (***) were considered statistically significant.

## Results

### Testing the pluripotent nature of hESCs and hiPSCs

**Figure 1:**
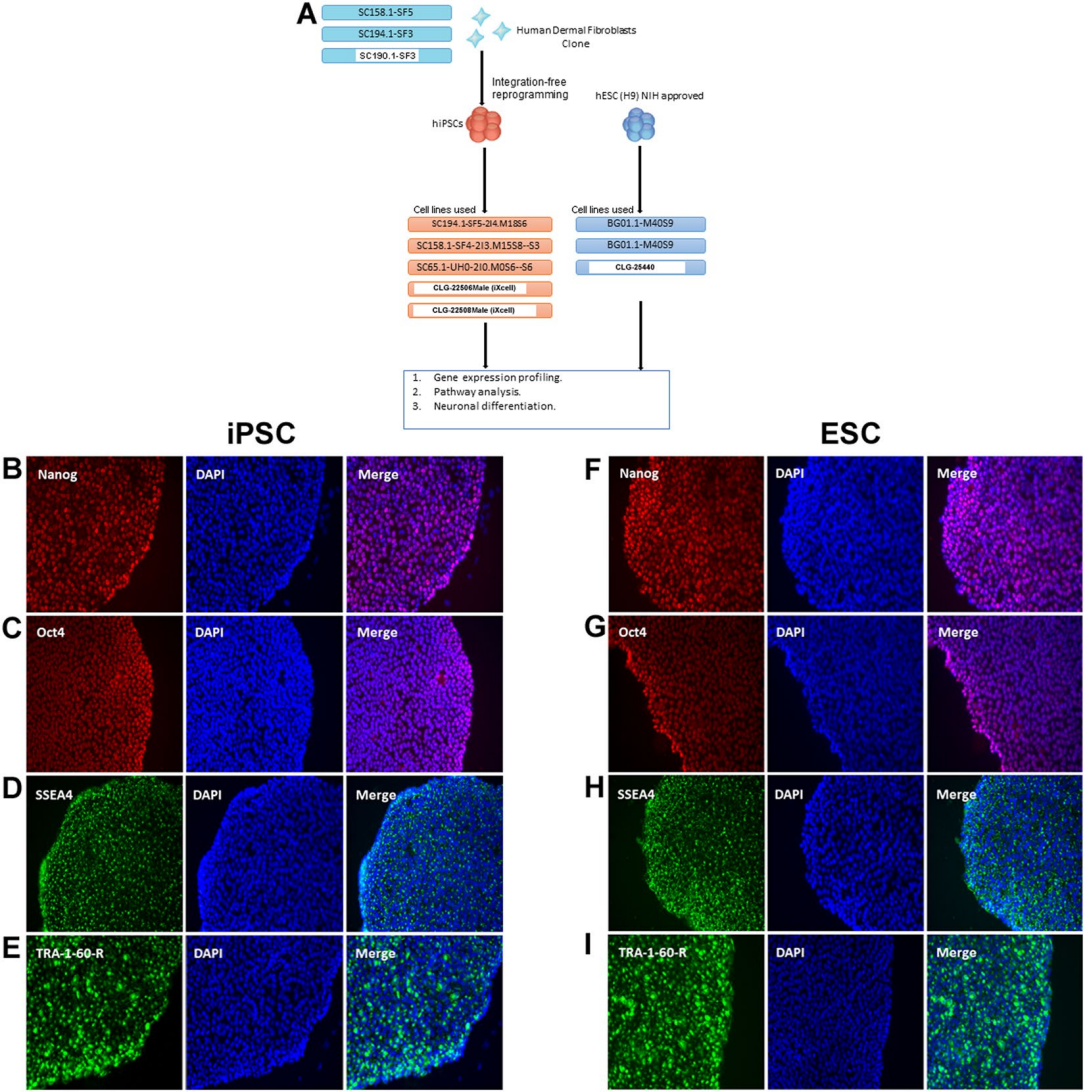
(**A**) Schematic overview for the experimental design used. (**B**–**I**) Immunostaining of pluripotency markers (Nanog, Oct4, SSEA4, TRA-1-60-R) in iPSC and in ESC.

An overview of the experimental design is given in Fig. 1A. We used genetically unmatched iPSC, and H9 ESC (Supplementary Table 1). To confirm the pluripotent nature of hESCs and hiPSCs, emerging colonies from both were picked up after ~3 weeks, proliferated, and proved to be positive for Nanog, Oct4, SSEA4, and TRA-1-60-R indicating successful reprogramming of iPSCs (Fig. 1B–I). The HDF were not reactive to any of the previously mentioned pluripotent markers (data not shown).

### Global gene expression changes among cell linesh

**Figure 2:**
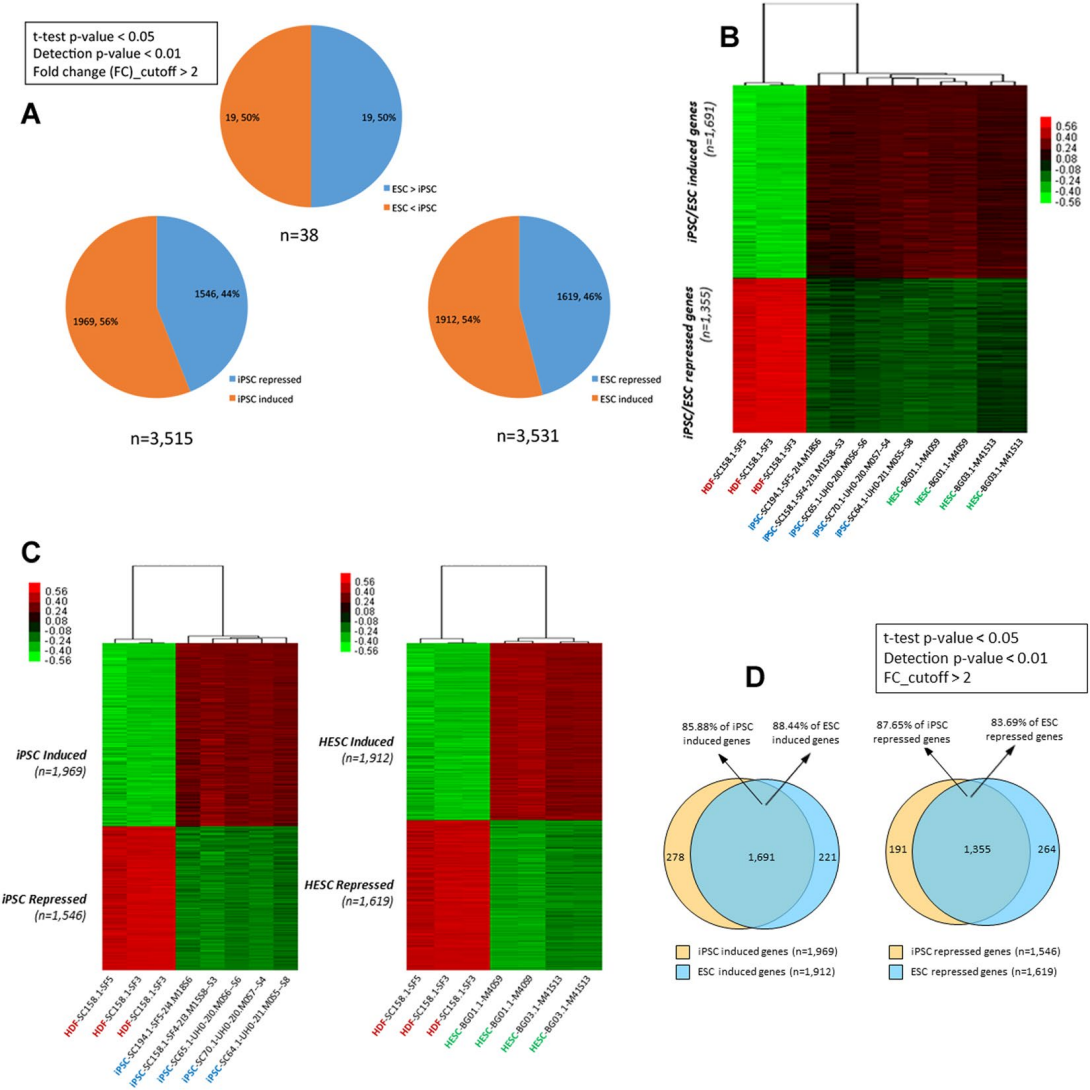
(**A**) Global gene expression changes among cell lines. (**B**) Hierarchical clustering and interclass correlations between iPSC/HESC and fibroblasts. (**C**) Hierarchical clustering and interclass correlations between iPSC/HESC and fibroblasts. (**D**) Common gene expression changes in human iPSC/ESC compared to fibroblasts.

Analysis of global gene expression using DNA microarray revealed a total of 3515 and 3513 dysregulated genes in iPSCs and hESCs, respectively (Fig. 2A). In hiPSCs, 1969 (56%) and 1546 (44%) genes were up- and downregulated, respectively, whereas in hESCs 1912 (54%) and 1619 (46%) genes were up- and downregulated, respectively (Fig. 2A).

### Hierarchical clustering and interclass correlations between hiPSC/hESC in comparison to fibroblasts

Using hierarchical clustering, we compared the expression profiles of up-and down regulated genes in hESCs, hiPSCs, and HDF. A clear segregation was noticed between the pluripotent cells and differentiated fibroblasts (Fig. 2B), indicating distinct genetic states. Careful examination of the heat maps indicated distinct differences between hESCs, hiPSCs on one side, and HDF on the other side (Fig. 2B). In contrast, the genetic profiles of hiPSCs and hESCs were clearly similar but not identical (Fig. 2B,C). The majority of the genes induced by both hiPSCs and hESCs were in common (n = 1691, 88.9% of induced in hiPSCs and 88.4% of induced in hESCs). Similarly, 1355 repressed genes (87.7% of hiPSCs and 83.7% of hESCs) were in common (Fig. 2D) This indicated similar but not identical genetic states between hiPSCs and hESCs. The observed segregation concurred in the genetic background of each cell line with distinct variations between the gene expression profile of HDF and pluripotent hiPSCs and hESCs. That is, hESC and hiPSC clones generated by non-integrating protocol clustered together, whereas HDF clones formed a distinct cluster group quite distant from the pluripotent clones clusters (Fig. 2B,C).

Distinctive sets of 278, and 221 induced genes are exclusive to hiPSC and hESC, respectively (Fig. 2D). Likewise, distinctive sets of 191, and 264 repressed genes are exclusive to hiPSC and hESC, respectively (Fig. 2D).

### Gene ontology (GO) analysis (Biological Process) of differentially expressed genes between hiPSCs/hESCs and human dermal fibroblasts

Although both hiPSCs and hESCs were separately clustered from HDFs, analysis of gene expression profile between the two pluripotent population suggests the presence of subtle but consistent gene expression profile differences (Fig. 2B).

**Figure 3:**
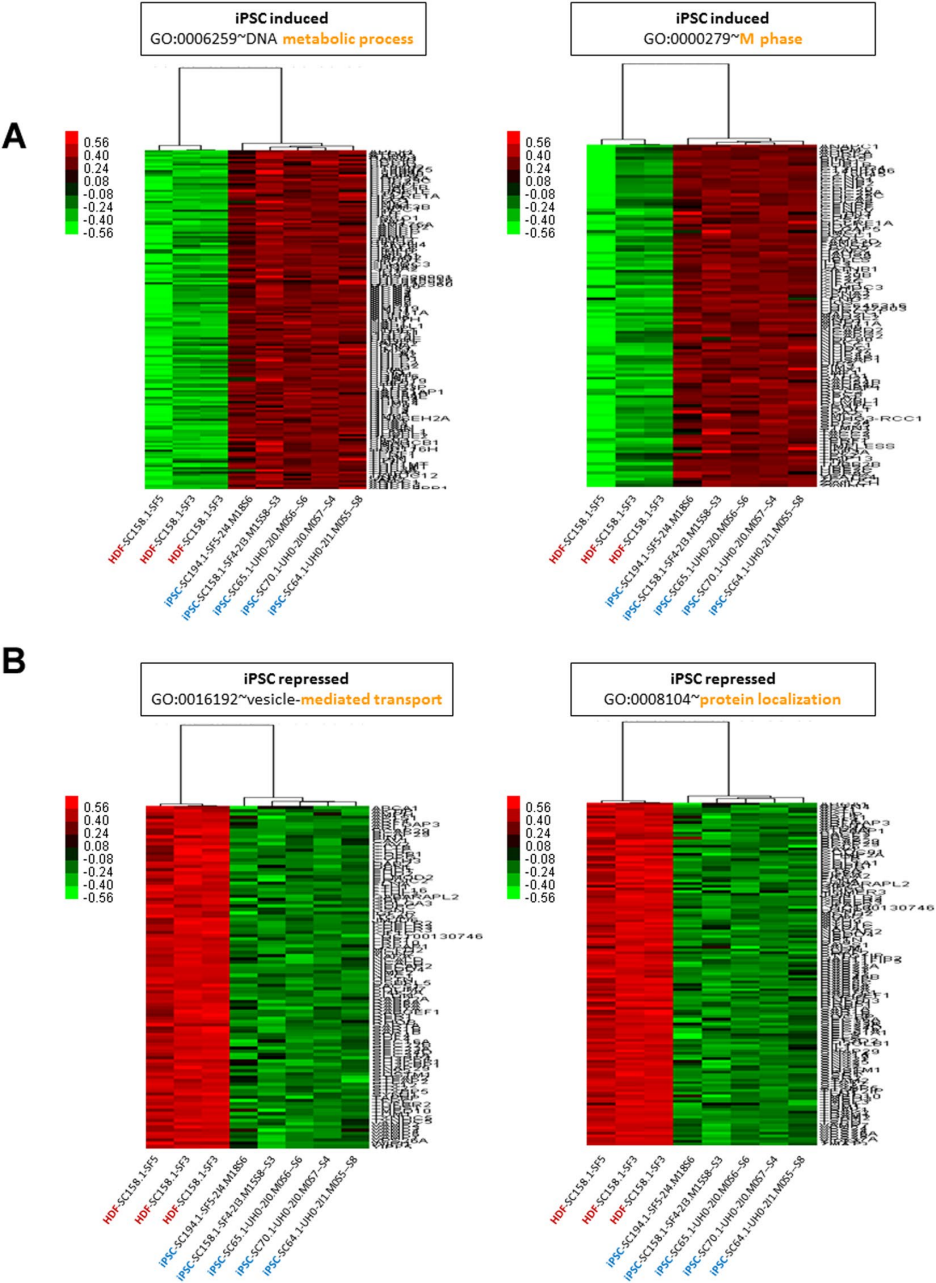
(**A**) Expression patterns of upregulated genes from the top two GO clusters (BP) of human iPSCs. (**B**) Expression patterns of downregulated genes from the top two GO clusters (BP) of human iPSCs.

**Figure 4:**
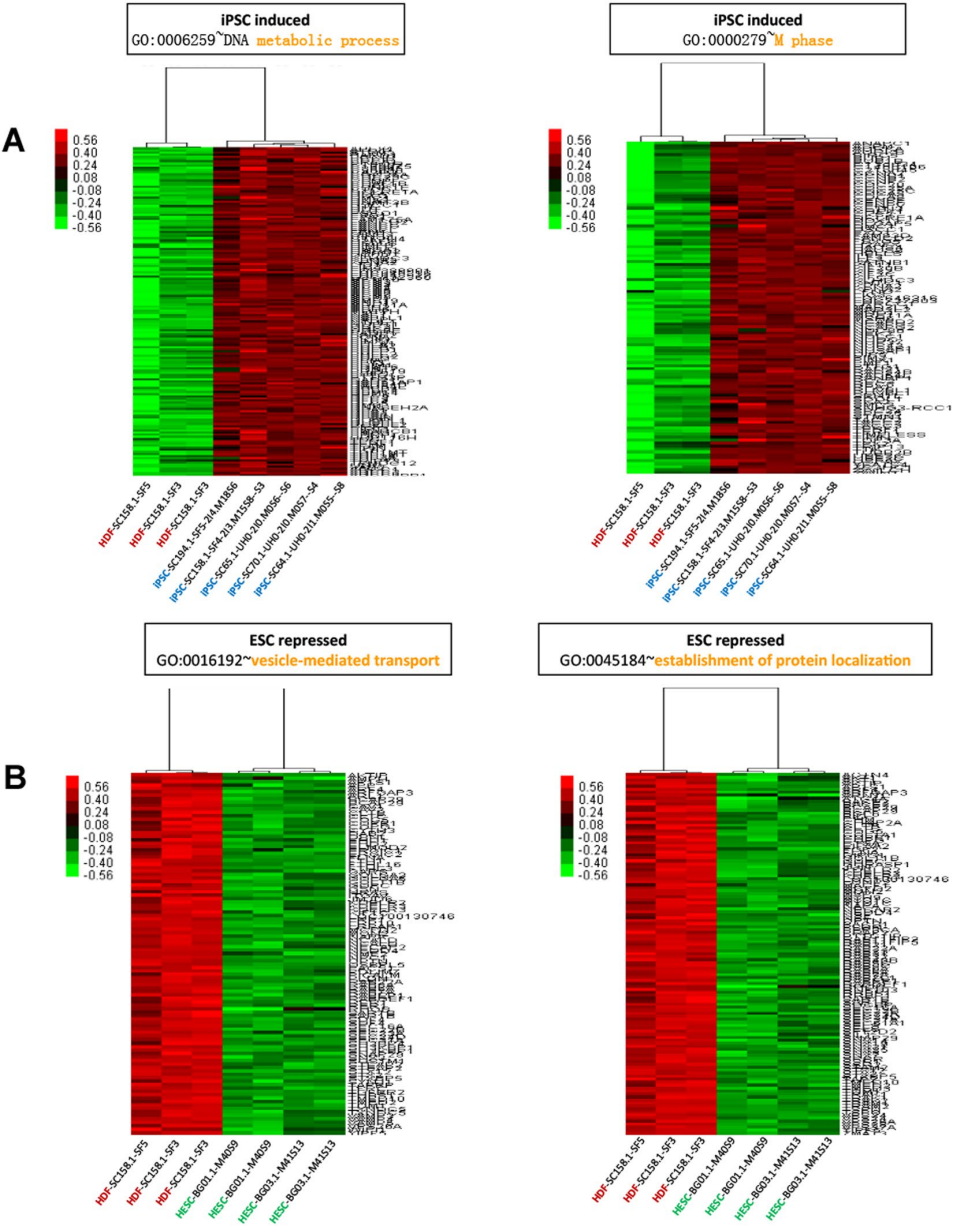
(**A**) Expression patterns of upregulated genes from the top two GO clusters (BP) of human ESCs. (**B**) Expression patterns of downregulated genes from the top two GO clusters (BP) of human ESCs.

To examine the effect of minor differences in the gene expression profile between hiPSCs and hESCs, we examined the Gene Ontology (GO) enrichment of hiPSCs and hESCs in comparison to HDFs. We detected a number of Biological Processes (BPs) enriched by the genes upregulated in hiPSCs of which the DNA metabolic process (GO:0006259; 140 genes; p = 1.94 × 10^−37^), and M phase (GO:0000279; 103 genes; p = 2.37 × 10^−32^) were the most enriched ones (Supplementary Table 2, Fig. 3A). Likewise, for the down-regulated genes in hiPSCs, we observed enrichment of BPs such as vesicle-mediated transport (GO:0016192; 91 genes; p = 1.2 × 10^−13^), and protein localization (GO:0008104; 141 genes; p = 5.6 × 10^−11^) were significantly enriched (Supplementary Table 2, Fig. 3B). GO analysis (BP) of genes upregulated in hESCs revealed significant enrichment of GO terms including DNA metabolic process (GO:0006259; 127 genes; p = 1.12 × 10^−31^), and M phase (GO:0000279; 95 genes; 1.26 × 10^−28^) (Supplementary Table 3, Fig. 4A). GO analysis (BP) of the genes downregulated in hESCs revealed significant enrichment of a number of BPs including vesicle-mediated transport (GO:0016192; 92 genes; p = 1.49 × 10^−12^), and establishment of protein localization (GO:0045184; 105 genes; p = 5.07 × 10^−10^) (Supplementary Table 3, Fig. 4B).

### Gene ontology (GO) analysis (Biological Process) of the common induced genes in human iPSCs/ESCs lines

**Figure 5:**
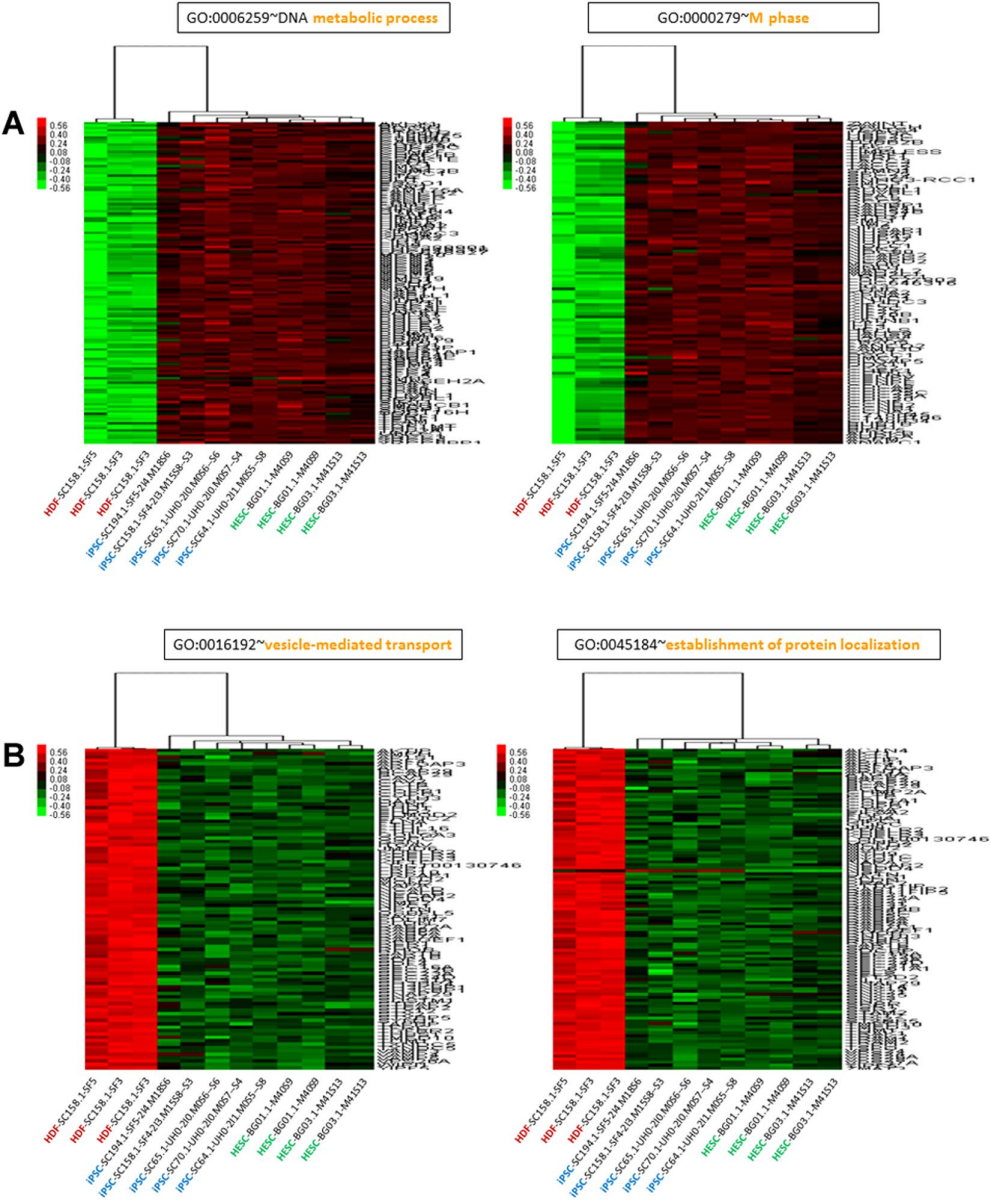
(**A**) GO clusters (BP) of common induced genes in human iPSCs/ESCs. (**B**) GO clusters (BP) of common repressed genes in human iPSCs/ESCs.

GO analysis (BP) of the genes induced in both iPSCs/hESCs lines revealed significant enrichment of several GO terms (Supplementary Table 4, Fig. 5A), of which the DNA metabolic process (GO:0006259; 123 genes; p = 3.5 × 10^−33^), and M phase (GO:0000279; 94 genes; p = 4.54 × 10^−31^) were the most significant.

BPs enriched by the genes repressed in both human iPSC/ESC lines included vesicle-mediated transport (GO:0016192; 82 genes; p = 1.54 × 10^−12^), and establishment of protein localization (GO:0045184; 95 genes; p = 7.6 × 10^−11^) (Supplementary Table 5, Fig. 5B).

### Pathways altered by dysregulated genes in human iPSCs and ESCs

Several KEGG pathways were enriched by the genes upregulated in hiPSCs (Supplementary Table 6), including DNA replication (hsa03030; 24 genes; p = 9.98 × 10^−17^), and Spliceosome (hsa03040; 44 genes; p = 1.36 × 10^−16^). GO analysis (Pathway) of downregulated genes in hiPSCs revealed enrichment in several KEGG pathways (Supplementary Table 6), of which Lysosome (hsa04142; 37 genes; p = 8.71 × 10^−13^), and Focal adhesion (hsa04510; 49 genes; 4.46 × 10^−12^) were the most significant.

Genes induced in hESCs also enriched pathways similar to that of hiPSC induced genes including the top KEGG pathways of DNA replication (hsa03030; 23 genes; p = 6.09 × 10^−16^), and Spliceosome (hsa03040; 38 genes; p = 8.96 × 10^−13^). Similar KEGG pathways were enriched with genes downregulated in hESCs as in hiPSCs (Supplementary Table 7).

Genes induced common to both hiPSCs and hESCs revealed enrichment in several KEGG pathways (Supplementary Table 8), of which DNA replication (hsa03030; 23 genes; p = 1.06 × 10^−16^) and Spliceosome (hsa03040; 36 genes; p = 2.08 × 10^−12^) were the most significant. KEGG pathways significantly enriched by repressed genes common to hiPSCs and hESCs included Focal adhesion (hsa04510; 46 genes; p = 3.32 × 10^−12^) and Lysosome (hsa04142; 32 genes; 1.08 × 10^−10^) (Supplementary Table 9).

### Functional consequences of differentially expressed genes (DEGs) in hiPSCs and hESCs

Next we explored the potential functional consequences of the differentially expressed genes (DEGs) in iPSCs and ESCs. We focused on induced/repressed genes characteristic for each cell type: 278 induced and 191 repressed genes for hiPSCs, and 221 induced and 264 repressed genes for hESCs. We used DAVID to analyse enriched/suppressed pathways for induced/repressed gene list for each cell type (iPSCs and ESCs). KEGG pathway analysis for 278 hiPSCs induced genes revealed the enrichment of five pathways: Cell cycle (6 genes), Butanoate metabolism (3 genes), Base excision repair (3 genes), and Synthesis and degradation of ketone bodies (2 genes). In comparison, the KEGG pathway analysis for 221 repressed hiPSC genes revealed the enrichment of T cell receptor signaling pathway (2 genes).

Notably, pathway analysis for the 191 and 264 repressed genes in hiPSCs and hESCs respectively disclosed the enrichment of the same KEGG pathways that included Focal adhesion (16 genes), ECM-receptor interaction (8 genes), p53 signaling pathway (5 genes), Complement and coagulation cascades (4 genes), and Lysosome (5 genes).

Next we focused on a group of highly modulated genes of both iPSCs and hESCs as a means to explore potential functional differences between the two cell populations. We focused on two of the genes involved in energy metabolism; LDHA and SLC2A1 (also known as GLUT1), because of their previously reported strong basal expression in hESCs and reduced expression in all hiPSCs^24^. Our data did not reveal any modulation in the gene expression level of LDHA and SLC2A1 in hiPSCs/hESCs.

Low variability and high connectivity provide stability to the network, contributing to highly conserved core processes common to all members of a pluripotent cell population^25^. So we studied the potential differences in the gene expression profiles of pluripotent markers between hiPSCs and hESCs. We focused on key pluripotency regulators (POU5F1, DNMT3b, SOX2, DPPA4, LIN28, CLDN7, FGFR4, and ZFP42). No induction/repression was demonstrated in the expression levels in any of the selected pluripotency regulators, a finding that might indicate their stable expression and low variability in the strongly self-renewing fraction of hiPSCs and hESCs.

### Neural Progenitor Cells derived from iPSC/ESC

**Figure 6:**
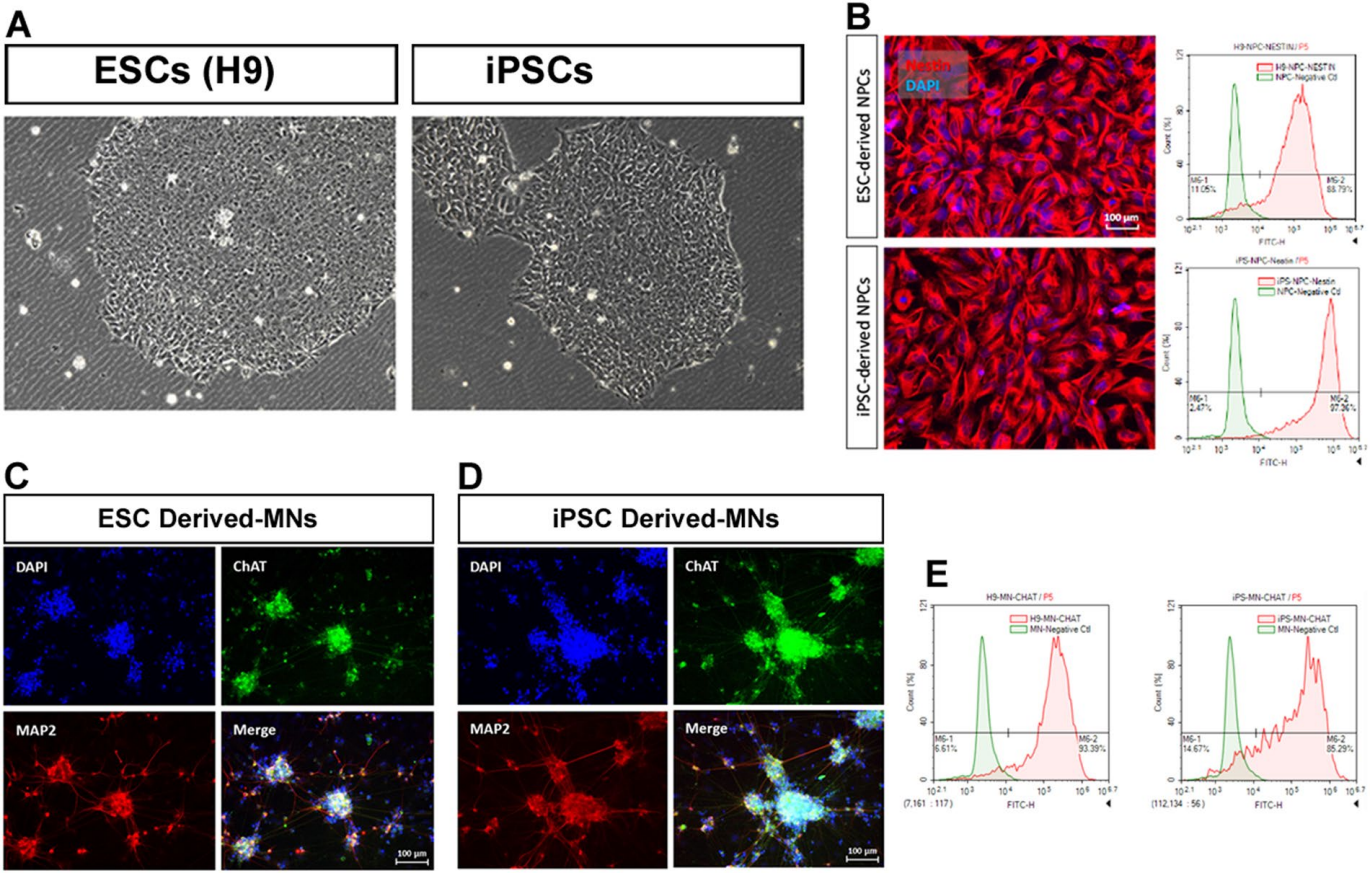
(**A**) H9 ESC and iPSC colonies in bright field. (**B**). Neural Progenitor Cells derived from iPSC/ESC. (**C**) Immunofluorescence Staining of ESC-derived MNs. (**D**) Immunofluorescence Staining of iPSC-derived MNs. Scale 100 µm. (**E**) Flow Cytometry.

The iPSC and H9 ESC were differentiated into NPCs and MNs *in vitro*. Each differentiation step were confirmed by immunostaining and flow cytometry (NPC: nestin; MN: CHAT). mRNA extracted from iPSC/ESC, NPCs, MNs were subjected to RT-PCR for the profiling of the top 10 induced/repressed genes in iPSC and ESC. ESCs (H9) and iPSCs were expanded using mTeSR™1 medium (STEMCELL Technologies) without feeder cells (Fig. 6A). The iPSC produced more NPC as compared to ESC where 97.36% and 88.79% of iPSC and ESC were differentiated into NESTIN-positive NPC, respectively (Fig. 6B). NPCs derived from ESCs or iPSCs were further differentiated into mature motor neurons using the proprietary protocol (iXCells Biotechnologies). The motor neurons (MNs) were characterized by immunofluorescence staining (ChAT, MAP2) (Fig. 6C,D) and flow analysis (Fig. 6E), and also collected for RNA extraction.

Motor Neurons (MNs) derived from iPSC and H9 embryonic stem cells were confirmed by immunostaining and flow cytometry using anti-ChAT antibody (Fig. 6C,D). 93.39% and 85.29% of ESC and iPSCS were differentiated into CHAT-positive motor neurons, respectively (Fig. 6E).

Based on the microarray analysis, the expression profile the top ten “Induced/upregulated” or “Repressed/downregulated” genes between iPSC and ESC were grouped. Primers are designed to amplify coding region of the listed genes (Supplementary Table 10).

For H9 ESC and iPSC, the relative expression of selected upregulated or downregulated genes was calculated based on the internal control housekeeping gene GAPDH, and then normalized to the ESCs, and iPSC, respectively.

**Figure 7:**
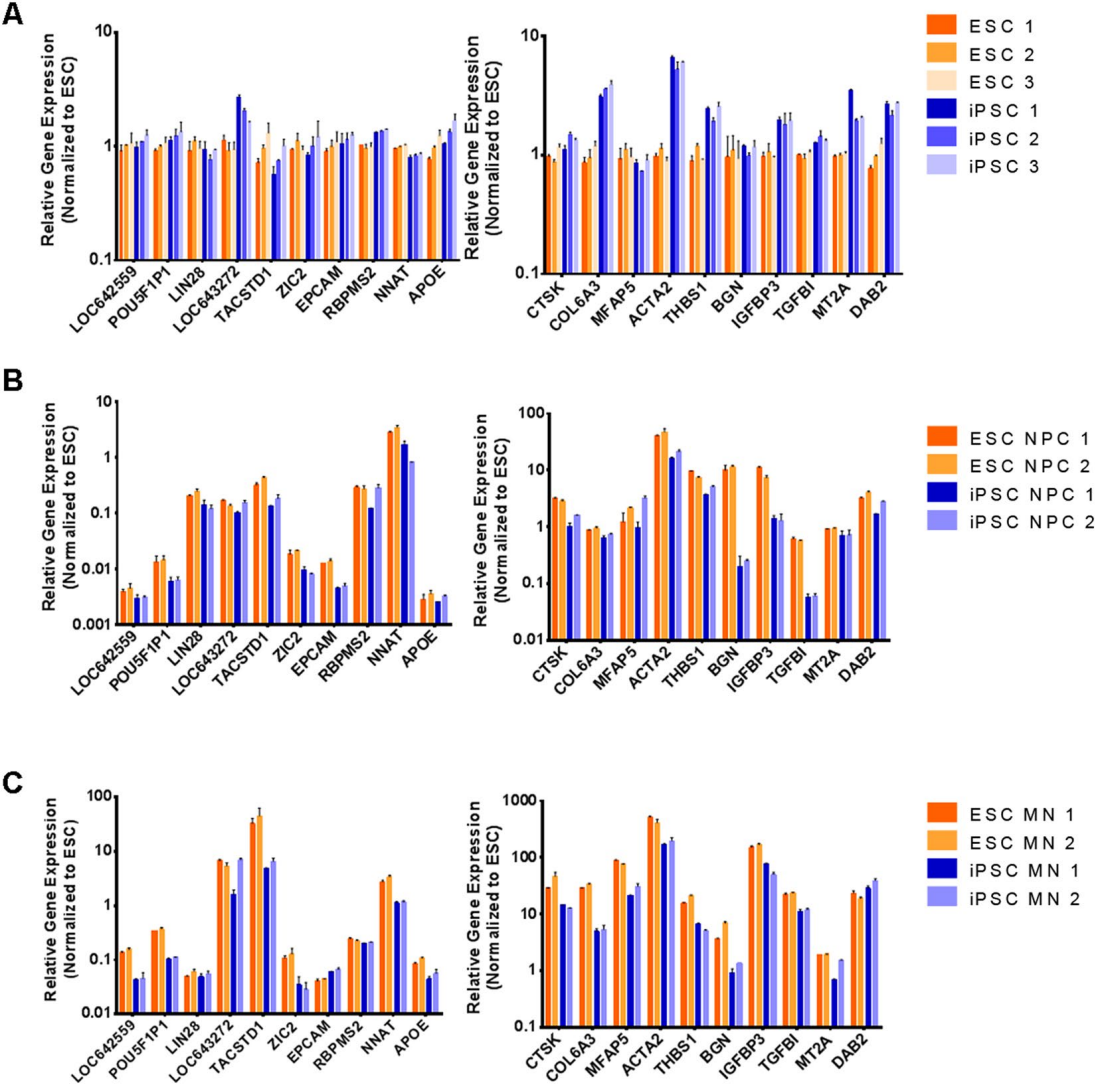
The relative expression of selected upregulated or downregulated genes in iPSCs and ESCs (**A**); NPCs derived from ESCs or iPSCs (**B**), and MNs derived from ESCs or iPSCs (**C**) was calculated based on the internal control housekeeping gene GAPDH, and then normalized to ESCs. The log transformed data was shown. n = 3. Error Bar: STDEV. y axis: Relative gene expression level normalized to ESCs. ESC: embryonic stem cells. iPSC: induced pluripotent stem cells.

For the H9 ESC, and iPSC upregulated genes (LOC642559, POU5F1P1, LIN28, LOC643272, TACSTD1, ZIC2, EPCAM, RBPMS2, NNAT, APOE), similar pattern of gene expression profile were identified during ESC and iPSC differentiation into NPC and MNs. The expression level of NNAT was significantly high in NPC, and MNs as compared to ESC, and iPSC. Also, the expression level of TACSTD1 was significant high in MNs as compared to ESC, and NPC (Fig. 7A,B). For the downregulated genes (CTSK, COL6A3, MFAP5, ACTA2, THBS1, BGN, IGFBP3, TGFBI, MT2A, and DAB2), all of them were significantly expressed in MNs as compared to ESC, and NPC (Fig. 7A–C).

For NMJ Function Assay (Contractility Assay), the C2C12 mouse myofibroblasts were differentiated into myotube and the iPSC-MNs or ESC-MNs were seeded on top of the differentiated myotubes. In both iPSC and H9 ESC, the MN-dependent contraction of myotubes was observed after 4 days of co-culture. The videos were recorded using Leica DMi8 microscope (Supplementary videos 1,2).

## Discussion

We studied the gene expression profile, pathways altered, and neuronal differentiation potential of hESCs and hiPSCs in order to identify possible equivalence between the two cell lines.

To compare the gene expression profiles of hESCs and hiPSCs, we removed most of major confounding factors that are known to affect gene expression profiling and to alter biological pathways such as the transfections methods where we are used integration free reprogramming technique. We generated hiPSCs using a non-integrating reprogramming protocol in which human dermal fibroblasts (HDF) were transfected with episomal DNAs expressing Oct4, Sox2, Klf4, Nanog, L-Myc and shRNA against p53^26^. Episomal DNAs are difficult to integrate into the parental infected HDF, and are diluted with subsequent cellular replication. This effectively leaves reprogrammed iPSCs with no unique genetic footprint. For the hiPSCs, we used five cell lines that were generated from HDF to ensure uniformity of transcriptomes. This approach helps to ensure epigenetic and genetic homogeneity between parental HDF and the reprogrammed iPSCs derived from them. Having similar/uniform epigenetic signatures for HDF and iPSCs would help to understand and highlight the effects of potential epigenetic background differences between the hiPSCs and hESCs. That is one of the major confounding factors between the two pluripotent cell population.

In differential expression analysis and clustering, hiPSCs and hESCs were clearly segregated from HDFs indicating their distinct genetic background. Variables such as genetic background, and generation of iPSCs from a single donor have been reported to contribute to the magnification of genetic and epigenetic differences between hESCs and hiPSCs^24,27–29^. By using a moderate number of samples (n = 11), we identified subtle molecular differences between hiPSCs and hESCs. Although using a higher number of samples might have minimized most of the previously mentioned variables, it might also contribute to loss of small but vital pathway differences^8^.

### Common induced and repressed genes in hiPSC and hESC lines

The great majority of modulated (induced/repressed) genes between undifferentiated hiPSC and hESC lines displayed a similar expression profile that were obviously linked to common biological processes and pathways. A set of common induced (1,691), and repressed (1,355) genes were identified in hiPSCs and hESCs lines. Such observation could indicate the close similarities between the hiPSCs and hESCs lines where supervised clustering could not distinguish hESCs from hiPSCs. Collectively, these data support the view that the genetic background didn’t account for the previously reported transcriptional differences between hESCs and hiPSCs. The common genetic pool of induced/repressed genes between hiPSCs and hESCs could highlight the existence of a large degree of genetic matching between the two pluripotent cell lines. Moreover, the present results suggest that hESC and hiPSC lines are equivalent, and the remaining differentially expressed genes identified might represent transcriptional noise induced by other confounding factors but not the genetic background.

Our findings agree with previous studies which have identified similar sets of differentially expressed genes when comparing genetically unmatched hiPSCs and hESCs using a similar cutoff^1,2,30^. In genetically matched hiPSCs and hESCs, a smaller number of differentially expressed genes were demonstrated^24^. These findings indicate that most of differentially expressed genes identified in the present study could be due to the use of genetically unmatched hiPSCs and hESCs. Moreover, the distinct overlap of the vast majority of induced/repressed genes between our examined hiPSCs and hESCs might provide a further support to our notion regarding potential equivalency and genetic matching between them.

To further support our findings, no evidence for differences in functional outcome was identified after testing the potential functional outcomes of a group of deferentially expressed genes (LDHA, SLC2A1, IRX2 and DPP10) These genes are known to play crucial roles in energy production or differentiation potential in neural cells or embryoid bodies, between genetically matched hiPSCs and hESCs^24^. The undifferentiated nature of hiPSCs and hESCs makes it difficult to determine whether the absence of any functional outcome for induced/repressed genes is due to aberrant expression or defective post-transcriptional mechanisms, the latter appear to be the case with LDHA^24^.

We have demonstrated that the Gene Ontogeny (GO) analysis of the top induced genes in hiPSCs and hESCs shows a significant enrichment of DNA metabolic process and M phase for both cell lines. Likewise, GO analysis (BP) of the top downregulated genes in both stem cell lines revealed significant enrichment of vesicle-mediated transport and establishment of protein localization.

GO analysis (Pathway) of the upregulated genes in iPSCs revealed enrichment in hsa03030:DNA replication (24 genes), and hsa03040:Spliceosome (44 genes). GO analysis (Pathway) of top 20% of downregulated genes in hESCs revealed enrichment in s hsa04142:Lysosome (37 genes), and hsa04510:Focal adhesion (49 genes).

Notably, the KEGG pathway analysis for repressed genes in hiPSCs, and hESCs, respectively disclosed the enrichment of the same KEGG pathways. These findings further supports our notion regarding the gene expression profiles similarity between hiPSCs, and hESCs. The fact that genetically unmatched hESC and hiPSC lines exhibit equivalent differentiation potentials using either a directed or spontaneous differentiation paradigm^24^ further supports our interpretation regarding the similar genetic status for hiPSCs, and hESCs.

Our study results suggest that the use of non-integrating vectors for reprogramming of HDF into iPSCs may prevent the introduction of tumorigenic transcriptional alterations into iPSCs. These findings are in agreement with those of Ohnuki *et al*.^31^ who demonstrated that hiPSC lines generated with integrating viruses exhibit dramatic differences in expression, methylation and differentiation potential compared to hiPSC lines generated with non-integrating reprogramming systems^31^. Moreover, the genetic signature introduced by SeV infection did not separate hESCs from hiPSCs previously generated with retroviral or episomal vectors^24^.

Next we focused on a group of highly modulated genes between the iPSCs, and hESCs as a mean to explore potential functional differences between the two cell population. We focused on two of the genes involved in energy metabolism; LDHA and SLC2A1 (also known as GLUT1), because of their previously reported strong basal expression in hESCs and reduced expression in all hiPSCs^24^. Our data did not reveal any modulation in the gene expression level of LDHA and SLC2A1 in the examined hiPSCs/hESCs.

Low variability and high connectivity contribute to highly conserved core processes common to all members of pluripotent cell populations^25^. The potential differences in the gene we examined in the expression profiles of pluripotent markers between hiPSCs and hESCs focused on key pluripotency regulators (POU5F1, DNMT3b, SOX2, DPPA4, LIN28, CLDN7, FGFR4, and ZFP42). No induction/repression was demonstrated in the expression levels in any of these; a finding that might indicate their stable expression, and low variability in the strongly self-renewing fraction of hiPSCs and hESCs.

The great majority of modulated (induced/repressed) genes between undifferentiated hiPSC and hESC lines displayed a similar expression profile that were obviously linked to common biological processes and pathways. A large set of common induced and repressed genes were identified in hiPSCs and hESCs lines. Such observation could indicate the close similarities between the hiPSCs and hESCs lines. Taken together, the present study suggests that differences in the gene expression between genetically unrelated hESC and hiPSC lines are primarily driven by other factors than the genetic background. Collectively, these data support the view that other parameters, such as reprogramming method, or gender might account for the majority of previously reported differences in the gene expression profile between hESCs and hiPSCs^32^.

### Use of neural differentiation to prove potential similarities between iPSC/ESC

The ability of iPSC and ESC to differentiate into NESTIN-NPC and ChAT-MNs was comparable; 97.36% vs 88.79% for NPC, and 85.29% vs. 93.39% for MNs, respectively. This might indicate similar neuronal differentiation potential. Moreover, similar pattern of gene expression profile were identified during ESC and iPSC differentiation into NPC and MNs. For the ten upregulated genes (LOC642559, POU5F1P1, LIN28, LOC643272, TACSTD1, ZIC2, EPCAM, RBPMS2, NNAT, APOE), the expression level of NNAT was significant in NPC as compared to ESC, iPSC, and MNs. Neuronatin (NNAT) was first identified as a brain-specific gene crucial for brain development. Ca2 + signaling, glucose transport, insulin secretion, and inflammation modulated at different pathological conditions have been proposed to be governed by NNAT^33^. Whereas, the expression level of TACSTD1 was significant in MNs as compared to ESC, iPSC, and NPC. Tumor-associated calcium signal transducer (TACSTD1) is a stem/progenitor cell marker, which is also upregulated in several human carcinomas^34^. For the downregulated genes (CTSK, COL6A3, MFAP5, ACTA2, THBS1, BGN, IGFBP3, TGFBI, MT2A, and DAB2), all of them were significantly expressed in MNs as compared to iPSC, ESC, and NPC. The similar gene expression pattern during the differentiation of iPSC and ESC into NPC, and MNs as well as their similar functional assays as demonstrated by NMJ formation provide another evidence for their potential equivalence.

Altogether, our findings prove that there is close similarity between genetically unmatched hiPSCs derived from HDFs and the “golden standard” pluripotent hESCs. Although the detailed molecular mechanisms underpinning such similarity are not fully understood, our work points out the importance of reprograming methods and their effects on the genetic background of the parental cells. Moreover, we identified similar pattern of gene expression profile during their differentiation into NPC and MNs. Such findings are crucial for the potential use of iPSCs in cellular based therapy of different neurological and neurodegenerative diseases, and disease modeling in which generation of hiPSCs using non-integrating programming methods from genetically unmatched iPSCs could be used for cellular based therapy; and in understanding the underpinning mechanisms associated with monogenic and polygenic diseases using genetically unmatched healthy and affected parental cell lines.

## Conclusion

We studied the differences between gene expression, molecular pathways, and motor cholinergic neurons differentiation potential between genetically unmatched hESC and hiPSC in order to highlight potential similarities/equivalence between the two cell lineages. At the gene expression and pathway level, hESC and hiPSC were very similar, but not identical. Pathway analysis of differentially expressed genes and assessment of cholinergic motor neurons differentiation potential disclosed not only enrichment of the same molecular pathways but also similar potential for differentiation into motor cholinergic neurons. Collectively, our data indicate that hESCs and hiPSCs are equivalent at the gene expression level, and their ability to differentiate into NPSCs and MNs, and that different genetic background of hiPSCs and hESCs does not confound their gene expression and neural differentiation potential.

## Electronic supplementary material

Video 1

Video 2

Supplementary Information
