# Supplementary material for: Genetically unmatched human iPSC and ESC exhibit equivalent gene expression and neuronal differentiation potential
Source: Sci Rep. 2017 Dec 13;7:17504. doi: 10.1038/s41598-017-17882-1 (PMC5727499; doi:10.1038/s41598-017-17882-1)
Supplement: Supplementary file 3 — Supplementary Information [file 41598_2017_17882_MOESM3_ESM.pdf]

Genetically unmatched human iPSC and ESC exhibit equivalent gene expression and neuronal differentiation potential

Hany E. Marei[1,] Althani A[1,2] *Lashen S* [3,] *Cenciarelli C*[4] *and Anwarul Hasan*[5]

[1] *Biomedical Research Center, Qatar University, Doha, PO Box 2713, Qatar,* [2]*Department of Health Sciences, College of Health Sciences, Qatar University, Doha 2713, Qatar,* [3]*Department of Cytology and Histology, Faculty of Veterinary Medicine, Mansoura University, Mansoura, Egypt,* [4]*Institute of Translational Pharmacology-CNR, Roma, Italy,* [5]*Department of Mechanical and Industrial Engineering, Qatar University, Doha, Qatar.*

Supplementary Tables

Supplementary Table 1: Human iPSC/ESC and dermal fibroblast RNA samples used in the microarray analysis.

Cell Line	Cell Type	RNA ng/ul	Group
SC194.1-SF5-2I4.M18S6	iPSC	701.34	Group 1 iPSC
SC158.1-SF4-2I3.M15S8--S3	iPSC	1860.65	Group 1 iPSC
SC65.1-UH0-2I0.M0S6--S6	iPSC	283.57	Group 1 iPSC
CLG-22506Male (iXcell)	iPSC	509.26	Group 1 iPSC
CLG-22508Male (iXcell)	iPSC	726.27	Group 1 iPSC
BG01.1-M40S9	HESC	313.19	Group 2 ESC
BG03.1-M41S13	HESC	321.22	Group 2 ESC
CLG-25440	HESC	303.30	Group 2 ESC
SC158.1-SF5	HDF	137.07	Control
SC194.1-SF3	HDF	150.47	Control
SC190.1-SF3	HDF	120.24	Control

Supplementary Table 2: Gene ontology (GO) analysis (Biological Process) of differentially expressed genes between iPSCs and fibroblasts.

Term	Count	%	P Value
GO:0006259~DNA metabolic process	140	8.70	1.94E-37
GO:0000279~M phase	103	6.40	2.37E-32
GO:0022403~cell cycle phase	117	7.27	3.92E-32
GO:0007049~cell cycle	167	10.37	3.30E-30
GO:0006260~DNA replication	74	4.60	4.63E-30
GO:0022402~cell cycle process	134	8.32	2.00E-28
GO:0000280~nuclear division	77	4.78	1.01E-27
GO:0007067~mitosis	77	4.78	1.01E-27
GO:0000087~M phase of mitotic cell cycle	77	4.78	3.83E-27
GO:0048285~organelle fission	77	4.78	1.92E-26

GO analysis (BP) of upregulated genes in iPSCs

Term	Count	%	P Value
GO:0016192~vesicle-mediated transport	91	6.93	1.20E-13
GO:0008104~protein localization	114	8.68	5.60E-11
GO:0045184~establishment of protein localization	103	7.84	7.51E-11
GO:0015031~protein transport	102	7.77	9.45E-11
GO:0048193~Golgi vesicle transport	32	2.44	8.99E-10
GO:0042127~regulation of cell proliferation	99	7.54	5.52E-09
GO:0051094~positive regulation of developmental process	48	3.66	8.62E-09
GO:0016044~membrane organization	59	4.49	8.93E-09
GO:0046907~intracellular transport	86	6.55	1.07E-08

GO:0007264~small GTPase mediated signal transduction	49	3.73	6.15E-08

GO analysis (BP) of downregulated genes in iPSCs

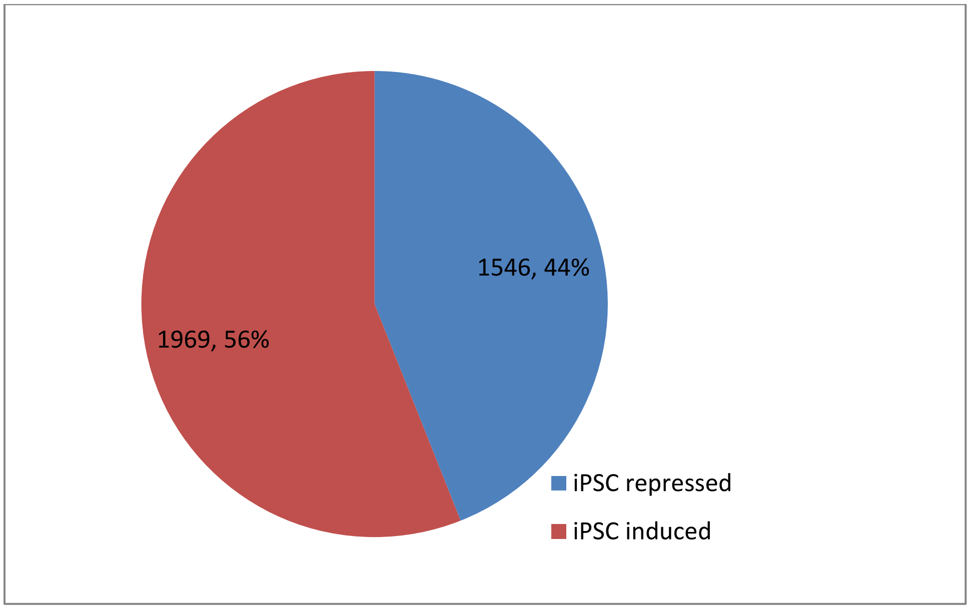

n=3,515

Supplementary Table 3: Gene ontology (GO) analysis (Biological Process) of differentially expressed genes between human ESCs and fibroblasts.

**Term**	**Count**	**%**	**P Value**
GO:0006259~DNA metabolic process	127	8.20	1.12E-31
GO:0000279~M phase	95	6.14	1.26E-28
GO:0007049~cell cycle	157	10.14	7.52E-28
GO:0022403~cell cycle phase	106	6.85	3.60E-27
GO:0006260~DNA replication	68	4.39	1.73E-26
GO:0022402~cell cycle process	125	8.07	1.03E-25
GO:0000280~nuclear division	71	4.59	1.39E-24
GO:0007067~mitosis	71	4.59	1.39E-24
GO:0000087~M phase of mitotic cell cycle	71	4.59	4.57E-24

GO:0048285~organelle fission	71	4.59	1.93E-23

GO analysis (BP) of upregulated genes in hESCs

Term	Count	%	P Value
GO:0016192~vesicle-mediated transport	92	6.71	1.49E-12
GO:0045184~establishment of protein localization	105	7.66	5.07E-10
GO:0008104~protein localization	116	8.46	5.24E-10
GO:0015031~protein transport	104	7.59	6.27E-10
GO:0001944~vasculature development	47	3.43	5.88E-09
GO:0001568~blood vessel development	46	3.36	8.03E-09
GO:0042127~regulation of cell proliferation	102	7.44	1.52E-08
GO:0051270~regulation of cell motion	39	2.84	1.70E-08
GO:0051094~positive regulation of developmental process	49	3.57	2.01E-08
GO:0046907~intracellular transport	88	6.42	3.93E-08

GO analysis (BP) of downregulated genes in hESCs

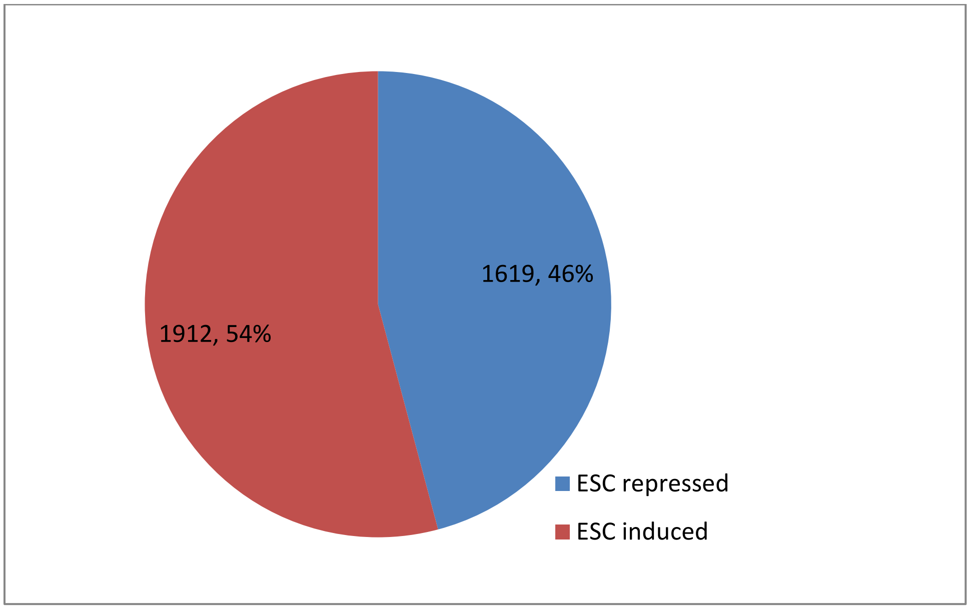

n=3,531

Supplementary Table 4: Gene ontology (GO) analysis (Biological Process) of the common induced genes in human iPSC/ESC lines.

Term	Count	%	P Value
GO:0006259~DNA metabolic process	123	8.86	3.50E-33
GO:0000279~M phase	94	6.77	4.54E-31
GO:0007049~cell cycle	153	11.02	2.38E-30
GO:0022403~cell cycle phase	105	7.56	6.46E-30
GO:0006260~DNA replication	68	4.90	7.21E-29
GO:0022402~cell cycle process	122	8.79	8.84E-28
GO:0000280~nuclear division	71	5.12	5.34E-27
GO:0007067~mitosis	71	5.12	5.34E-27
GO:0000087~M phase of mitotic cell cycle	71	5.12	1.81E-26
GO:0048285~organelle fission	71	5.12	7.95E-26

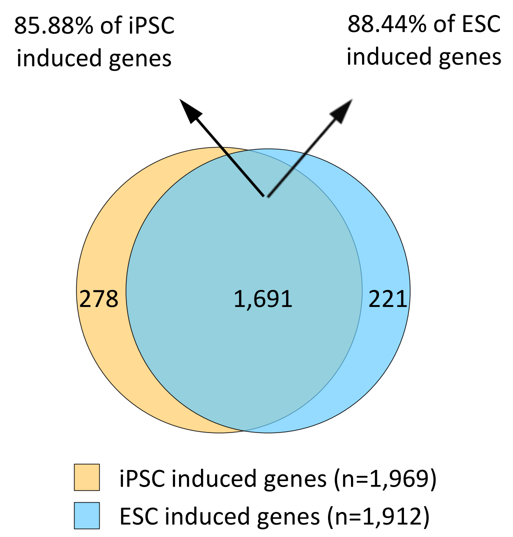

Supplementary Table 5: Gene ontology (GO) analysis (Biological Process) of the common repressed genes in human iPSC/ESC lines**.**

Term	Count	%	P Value
GO:0016192~vesicle-mediated transport	82	7.11	1.54E-12
GO:0045184~establishment of protein localization	95	8.23	7.60E-11
GO:0015031~protein transport	94	8.15	1.08E-10
GO:0008104~protein localization	104	9.01	1.30E-10
GO:0051094~positive regulation of developmental process	44	3.81	1.97E-08
GO:0007264~small GTPase mediated signal transduction	46	3.99	4.02E-08
GO:0046907~intracellular transport	77	6.67	6.23E-08
GO:0051270~regulation of cell motion	34	2.95	8.03E-08
GO:0048193~Golgi vesicle transport	27	2.34	9.19E-08
GO:0001568~blood vessel development	39	3.38	1.22E-07

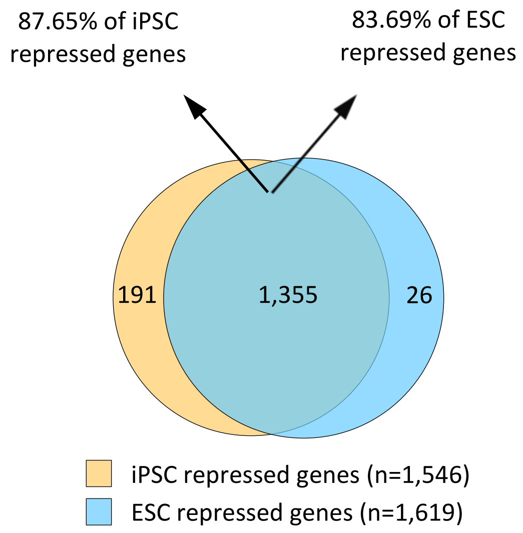

Supplementary Table 6: Gene ontology (GO) analysis (Pathway) of differentially expressed genes between iPSCs and fibroblasts.

Category	Term	Count	%	P Value
KEGG_PATHWAY	hsa03030:DNA replication	24	1.49	9.98E-17
KEGG_PATHWAY	hsa03040:Spliceosome	44	2.73	1.36E-16
KEGG_PATHWAY	hsa04110:Cell cycle	35	2.17	3.50E-10
KEGG_PATHWAY	hsa03410:Base excision repair	17	1.06	4.86E-09
KEGG_PATHWAY	hsa03430:Mismatch repair	13	0.81	6.75E-08
KEGG_PATHWAY	hsa03018:RNA degradation	17	1.06	1.20E-05
KEGG_PATHWAY	hsa00280:Valine, leucine and isoleucine degradation	13	0.81	2.02E-04
KEGG_PATHWAY	hsa03420:Nucleotide excision repair	13	0.81	2.02E-04
KEGG_PATHWAY	hsa00100:Steroid biosynthesis	8	0.50	2.75E-04
KEGG_PATHWAY	hsa00970:Aminoacyl-tRNA biosynthesis	12	0.75	4.34E-04

GO analysis (Pathway) of upregulated genes in iPSCs

Category	Term	Count	%	P Value
KEGG_PATHWAY	hsa04142:Lysosome	37	2.82	8.71E-13
KEGG_PATHWAY	hsa04510:Focal adhesion	49	3.73	4.46E-12
KEGG_PATHWAY	hsa00531:Glycosaminoglycan degradation	9	0.69	1.57E-04
KEGG_PATHWAY	hsa05212:Pancreatic cancer	17	1.29	1.91E-04
KEGG_PATHWAY	hsa04512:ECM-receptor interaction	18	1.37	4.00E-04
KEGG_PATHWAY	hsa04144:Endocytosis	30	2.28	4.57E-04
KEGG_PATHWAY	hsa04810:Regulation of actin cytoskeleton	32	2.44	0.001376
KEGG_PATHWAY	hsa05223:Non-small cell lung cancer	12	0.91	0.003947
KEGG_PATHWAY	hsa04722:Neurotrophin signaling pathway	20	1.52	0.005869

KEGG_PATHWAY	hsa04670:Leukocyte transendothelial migration	19	1.45	0.007578

GO analysis (Pathway) of downregulated genes in iPSCs

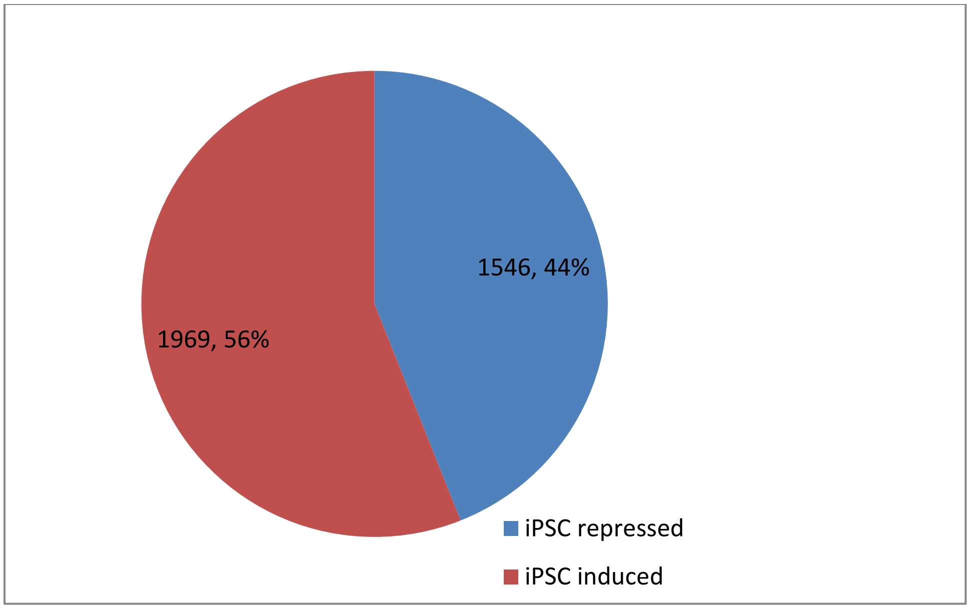

n=3,515

Supplementary Table 7: Gene ontology (GO) analysis (Pathway) of differentially expressed genes between human ESCs and fibroblasts.

Category	Term	Count	%	P Value
KEGG_PATHWAY	hsa03030:DNA replication	23	1.49	6.09E-16
KEGG_PATHWAY	hsa03040:Spliceosome	38	2.45	8.96E-13
KEGG_PATHWAY	hsa04110:Cell cycle	34	2.20	3.78E-10
KEGG_PATHWAY	hsa03410:Base excision repair	15	0.97	1.84E-07
KEGG_PATHWAY	hsa03430:Mismatch repair	12	0.78	4.45E-07
KEGG_PATHWAY	hsa00230:Purine metabolism	27	1.74	1.76E-04
KEGG_PATHWAY	hsa00100:Steroid biosynthesis	8	0.52	1.98E-04
KEGG_PATHWAY	hsa03420:Nucleotide excision repair	12	0.78	5.35E-04
KEGG_PATHWAY	hsa00280:Valine, leucine and isoleucine degradation	12	0.78	5.35E-04
KEGG_PATHWAY	hsa04114:Oocyte meiosis	20	1.29	0.001062

GO analysis (Pathway) of upregulated genes in ESCs

Category	Term	Count	%	P Value
KEGG_PATHWAY	hsa04510:Focal adhesion	50	3.65	3.06E-12
KEGG_PATHWAY	hsa04142:Lysosome	32	2.33	4.37E-09
KEGG_PATHWAY	hsa04144:Endocytosis	32	2.33	1.35E-04
KEGG_PATHWAY	hsa04512:ECM-receptor interaction	19	1.39	1.76E-04
KEGG_PATHWAY	hsa05212:Pancreatic cancer	16	1.17	8.28E-04
KEGG_PATHWAY	hsa00531:Glycosaminoglycan degradation	8	0.58	0.001218
KEGG_PATHWAY	hsa04810:Regulation of actin cytoskeleton	32	2.33	0.002084
KEGG_PATHWAY	hsa04722:Neurotrophin signaling pathway	21	1.53	0.003509
KEGG_PATHWAY	hsa04210:Apoptosis	16	1.17	0.005804

KEGG_PATHWAY	hsa05210:Colorectal cancer	15	1.09	0.010211

GO analysis (Pathway) of downregulated genes in ESCs

Category	Term	Count	%	P Value
KEGG_PATHWAY	hsa03030:DNA replication	23	1.49	6.09E-16
KEGG_PATHWAY	hsa03040:Spliceosome	38	2.45	8.96E-13
KEGG_PATHWAY	hsa04110:Cell cycle	34	2.20	3.78E-10
KEGG_PATHWAY	hsa03410:Base excision repair	15	0.97	1.84E-07
KEGG_PATHWAY	hsa03430:Mismatch repair	12	0.78	4.45E-07
KEGG_PATHWAY	hsa00230:Purine metabolism	27	1.74	1.76E-04
KEGG_PATHWAY	hsa00100:Steroid biosynthesis	8	0.52	1.98E-04
KEGG_PATHWAY	hsa03420:Nucleotide excision repair	12	0.78	5.35E-04
KEGG_PATHWAY	hsa00280:Valine, leucine and isoleucine degradation	12	0.78	5.35E-04
KEGG_PATHWAY	hsa04114:Oocyte meiosis	20	1.29	0.001062

GO analysis (Pathway) of upregulated genes in ESCs

Category	Term	Count	%	P Value
KEGG_PATHWAY	hsa04510:Focal adhesion	50	3.65	3.06E-12
KEGG_PATHWAY	hsa04142:Lysosome	32	2.33	4.37E-09
KEGG_PATHWAY	hsa04144:Endocytosis	32	2.33	1.35E-04
KEGG_PATHWAY	hsa04512:ECM-receptor interaction	19	1.39	1.76E-04
KEGG_PATHWAY	hsa05212:Pancreatic cancer	16	1.17	8.28E-04
KEGG_PATHWAY	hsa00531:Glycosaminoglycan degradation	8	0.58	0.001218
KEGG_PATHWAY	hsa04810:Regulation of actin cytoskeleton	32	2.33	0.002084
KEGG_PATHWAY	hsa04722:Neurotrophin signaling pathway	21	1.53	0.003509
KEGG_PATHWAY	hsa04210:Apoptosis	16	1.17	0.005804

KEGG_PATHWAY	hsa05210:Colorectal cancer	15	1.09	0.010211

GO analysis (Pathway) of downregulated genes in ESCs

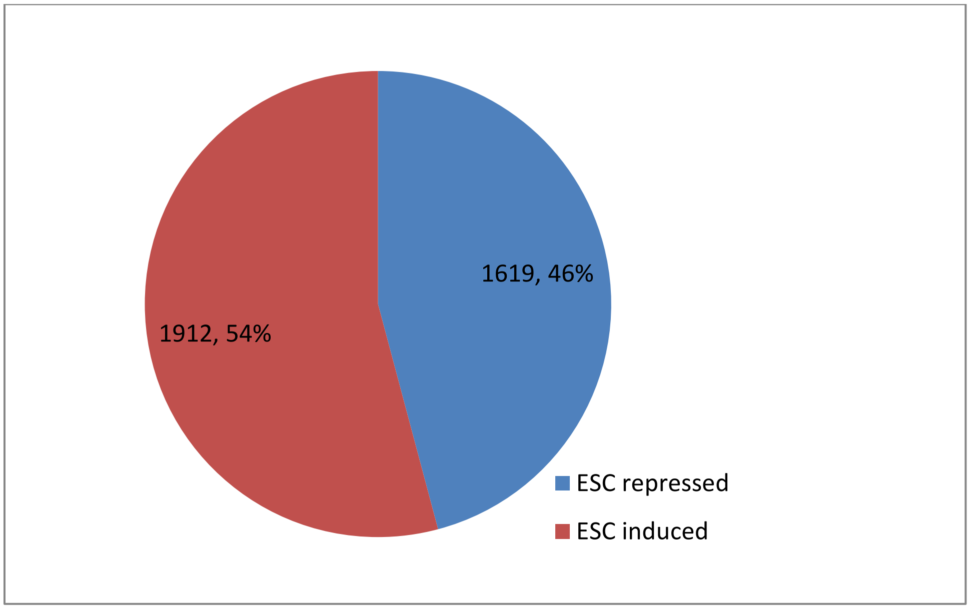

n=3,531

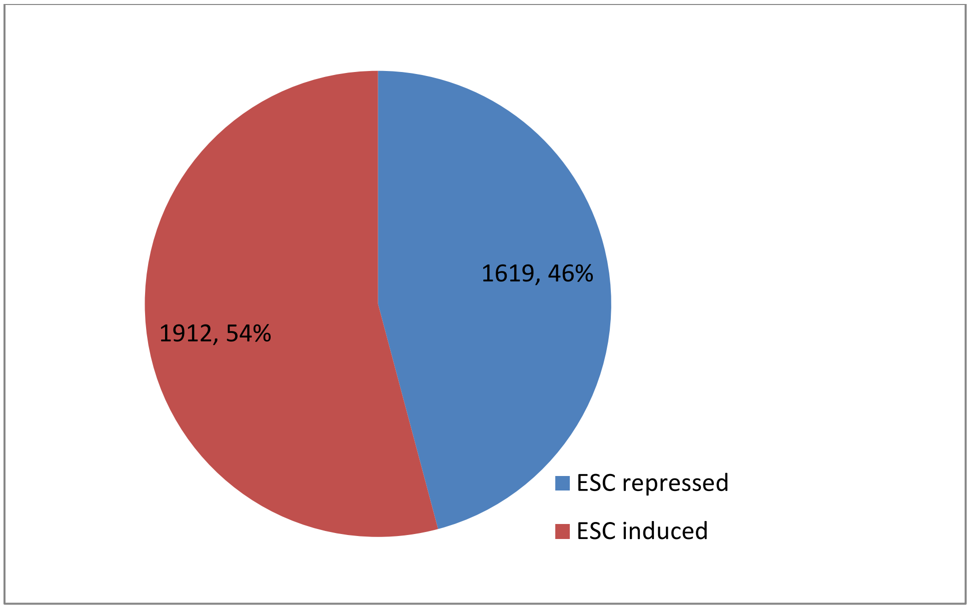

n=3,531

Supplementary Table 8: Gene ontology (GO) analysis (Pathway) of common induced genes in human iPSCs/ESCs.

Category	Term	Count	%	PValue
KEGG_PATHWAY	hsa03030:DNA replication	23	1.66	1.06E-16
KEGG_PATHWAY	hsa03040:Spliceosome	36	2.59	2.08E-12
KEGG_PATHWAY	hsa04110:Cell cycle	33	2.38	2.02E-10
KEGG_PATHWAY	hsa03410:Base excision repair	15	1.08	6.58E-08
KEGG_PATHWAY	hsa03430:Mismatch repair	12	0.86	1.93E-07
KEGG_PATHWAY	hsa00100:Steroid biosynthesis	8	0.58	1.18E-04
KEGG_PATHWAY	hsa00280:Valine, leucine and isoleucine degradation	12	0.86	2.64E-04
KEGG_PATHWAY	hsa03420:Nucleotide excision repair	12	0.86	2.64E-04
KEGG_PATHWAY	hsa04114:Oocyte meiosis	20	1.44	3.88E-04
KEGG_PATHWAY	hsa00230:Purine metabolism	23	1.66	0.001831

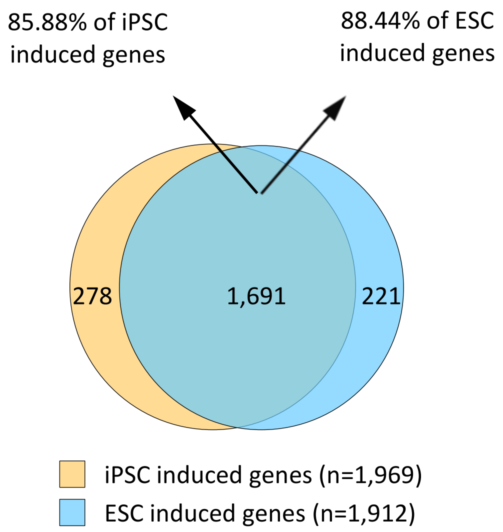

Supplementary Table 9: Gene ontology (GO) analysis (Pathway) of common repressed genes in human iPSCs/ESCs.

Category	Term	Count	%	P Value
KEGG_PATHWAY	hsa04510:Focal adhesion	46	3.99	3.32E-12
KEGG_PATHWAY	hsa04142:Lysosome	32	2.77	1.08E-10
KEGG_PATHWAY	hsa04144:Endocytosis	30	2.60	5.43E-05
KEGG_PATHWAY	hsa04512:ECM-receptor interaction	18	1.56	9.20E-05
KEGG_PATHWAY	hsa00531:Glycosaminoglycan degradation	8	0.69	5.01E-04
KEGG_PATHWAY	hsa05212:Pancreatic cancer	15	1.30	5.86E-04
KEGG_PATHWAY	hsa04810:Regulation of actin cytoskeleton	30	2.60	8.42E-04
KEGG_PATHWAY	hsa04670:Leukocyte transendothelial migration	18	1.56	0.005032
KEGG_PATHWAY	hsa05410:Hypertrophic cardiomyopathy (HCM)	14	1.21	0.008197
KEGG_PATHWAY	hsa04666:Fc gamma R-mediated phagocytosis	15	1.30	0.008595

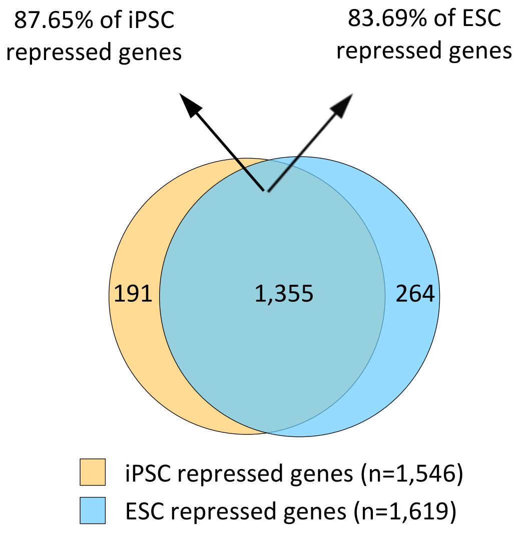

Supplementary Table 10: qRT-PCR Primers

	SYMBOL	SEARCH_KEY	Forward Primer Sequence	Reverse Primer Sequence
Induced	LOC642559	NM_203289.2	agcgatcaagcagtggcta	ccagaggaaaggacactggt
	POU5F1P1	NR_002304.1	gaaacccacactgcagatca	cggttacagaaccacactcg
	LIN28	NM_024674.3	ggttccagggagacacacaa	tactgtagcagggcaggaca
	LOC643272	XM_926633.1	cactgccggtttacactgttt	tttggggatagcaccaagag
	TACSTD1	NM_002354.1	gccaggtaaaagctcaaaggt	gaacgtggaggctaaaggaa
	ZIC2	NM_007129.2	cccagtcttctgacaaactgtgta	caaaaagcctttaaaaccacctc
	EPCAM	NM_002354.2	ccatgtgctggtgtgtgaa	tgtgttttagttcaatgatgatcca
	RBPMS2	NM_194272.1	tcacctacccaactgccact	ggtaccagcgcacctgag
	NNAT	NM_005386.2	gggtaggattcgcttttcg	aacacctcacttctcgcaatg
	APOE	NM_000041.2	ggtcgcttttgggattacct	catggtctcgtccatcagc
Repressed	CTSK	NM_000396.2	tgctgcatacacacagaaacac	agaaggaagggtagggagga
	COL6A3	NM_057164.2	gtgttctcggtgagcacctt	aggtcagggtcgtgatgg
	MFAP5	NM_003480.2	ggacccaaaatgtcttctgct	agttctatagctggcatagtcctca
	ACTA2	NM_001613.1	caccatcggaaatgaacgttt	gactccatcccgatgaagga
	THBS1	NM_003246.2	tgacaacaatcaggatattgatga	ttgggcacatagggacagtt
	BGN	NM_001711.3	ctcgtcctggtgaacaacaa	caggtggttcttggagatgtag
	IGFBP3	NM_001013398.1	actgaggaaaggagctcacg	actttgccagaccttcttgg
	TGFBI	NM_000358.1	cgagtgctgtcctggatatg	cccagggtctcgtaaaggtt
	MT2A	NM_005953.2	aacctgtcccgactctagcc	gcaggtgcaggagtcacc
	DAB2	NM_001343.1	tgagtgcctttgccagttatt	gcatcaaagtcatcatggtctg

Supplementary videos 1,2: C2C12 mouse myofibroblasts were seeded on 96-well plates in DMEM with 10% FBS, and myotube differentiation were initiated by switching to DMEM with 2% FBS for 3-4 days. The ESC-MNs (A) and iPSC-MNs (B) or were seeded on top of the differentiated myotubes with Motor Neuron Maintenance Medium. The MN-dependent contraction of myotubes was observed after 4 days of co-culture in both cell types. The videos were recorded using Leica DMi8 microscope.
